# Successful palliative hypofractionated radiotherapy with superficial hyperthermia for heavily pretreated cutaneous breast cancer lesions

**DOI:** 10.1007/s00066-026-02536-x

**Published:** 2026-06-22

**Authors:** D. M. V. de Vries-Huizing, A. Holtmaat, M. W. Kolff, J. Crezee

**Affiliations:** Department of Radiation Oncology, Amsterdam UMC, University of Amsterdam, Cancer Center Amsterdam, Meibergdreef 9, 1105 AZ Amsterdam, The Netherlands

**Keywords:** Skin metastases, Re-irradiation, Radiosensitization, Quality of Life, Superficial hyperthermia

## Abstract

**Background:**

Hypofractionated radiotherapy has been successfully shown to reduce pain and skin lesion bleeding. In previously irradiated areas, the effect of hypofractionated radiotherapy (weekly 4 Gy radiotherapy to a total dose of 20 Gy) can be enhanced by hyperthermia: increasing temperatures in target areas up to 43 °C. Superficial hyperthermia in combination with radiotherapy is a proven and widely applied treatment for recurrent breast cancer and malignant melanoma. Literature on the use of radiotherapy to cause hemostasis of the skin is available; however, the role of hyperthermia in combination with radiotherapy for this indication is not yet described.

**Short communication:**

We present a 72-year old woman with a history of breast cancer with painful and bleeding cutaneous metastases in the right upper arm and right breast which had a large impact on her quality of life. The metastases progressed under T‑deruxtecan treatment, and she was referred for re-irradiation to control the bleeding skin. Hyperthermia was added as a tumor-selective radiosensitizer to the radiotherapy fractions to enhance irradiation. The main treatment goal was to reduce bleeding and itching of the cutaneous lesions. The patient was treated once a week with hyperthermia immediately followed by 4 Gy radiotherapy fractions for 5 consecutive weeks. Bleeding was already decreased during the treatment series and the skin healed within 4 weeks after therapy.

**Conclusion:**

Treatment with 5 × 4 Gy radiotherapy combined with hyperthermia sessions was a successful palliative therapy for bleeding cutaneous metastases. The treatment schedule was well-tolerated and the skin fully recovered within 1 month.

## Background

Cutaneous breast cancer metastases are more often found on the chest wall and abdomen, whereby manifestations on limbs are less common. The primary treatment for cutaneous metastases is surgery and chemotherapy; however, larger areas might also be controlled by radiotherapy [1]. Palliative hypofractionated radiotherapy had been shown to successfully reduce pain and bleeding of cutaneous tumor lesions from skin malignancies [2]. Good local control was also achieved in patients with nonmelanoma skin lesions, which was especially relevant for older patients without surgical options [3]. Palliative re-irradiation of a large area of the skin is common, and tools to enhance the effect of radiotherapy are desirable as the maximum re-irradiation dose must be limited to avoid side effects due to excessive cumulative radiotherapy dose.

Hyperthermia combined with radiotherapy has been shown to be an effective concurrent treatment for numerous types of cancer. An increase in local temperature up to 40–43 °C for the duration of 1 h improves the effect of radiotherapy in multiple ways [4]. Hyperthermia directly kills hypoxic tumor cells, inhibits DNA damage repair, increases perfusion resulting in improved oxygenation, and activates the immune system [5]. Re-irradiation in combination with hyperthermia is proven more effective compared to radiation alone for recurrent breast cancer [1, 6–8]. Clinical data on radiotherapy combined with hyperthermia for breast cancer show a strong dose–effect relationship: higher temperatures result in improved local control [9].

Hyperthermia can be applied to different tumor locations using applicators of different sizes which emit electromagnetic radiation resulting in the production of heat [4]. Antennas for superficial hyperthermia typically use a microwave frequency of 915 or 434 MHz to treat target volumes up to approximately 4 cm beneath the skin, which is suitable for (sub)cutaneous tumor lesions of different origins [10].

In this short communication, we present a case of a women referred for radiotherapy to control bleeding cutaneous breast cancer metastases in the right arm and right breast in a previously irradiated area. Local superficial hyperthermia was added to the treatment to enhance the radiotherapy effect.

## Short communication

**Fig. 1:**
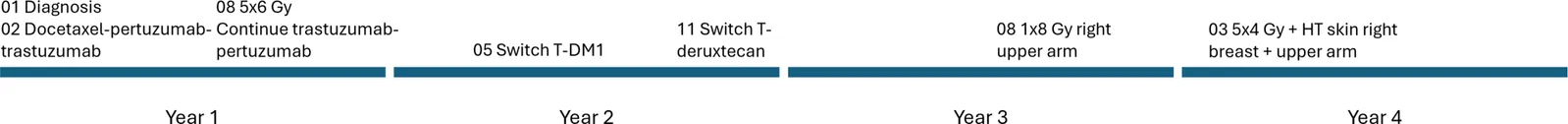
Treatment courses over time

**Fig. 2:**
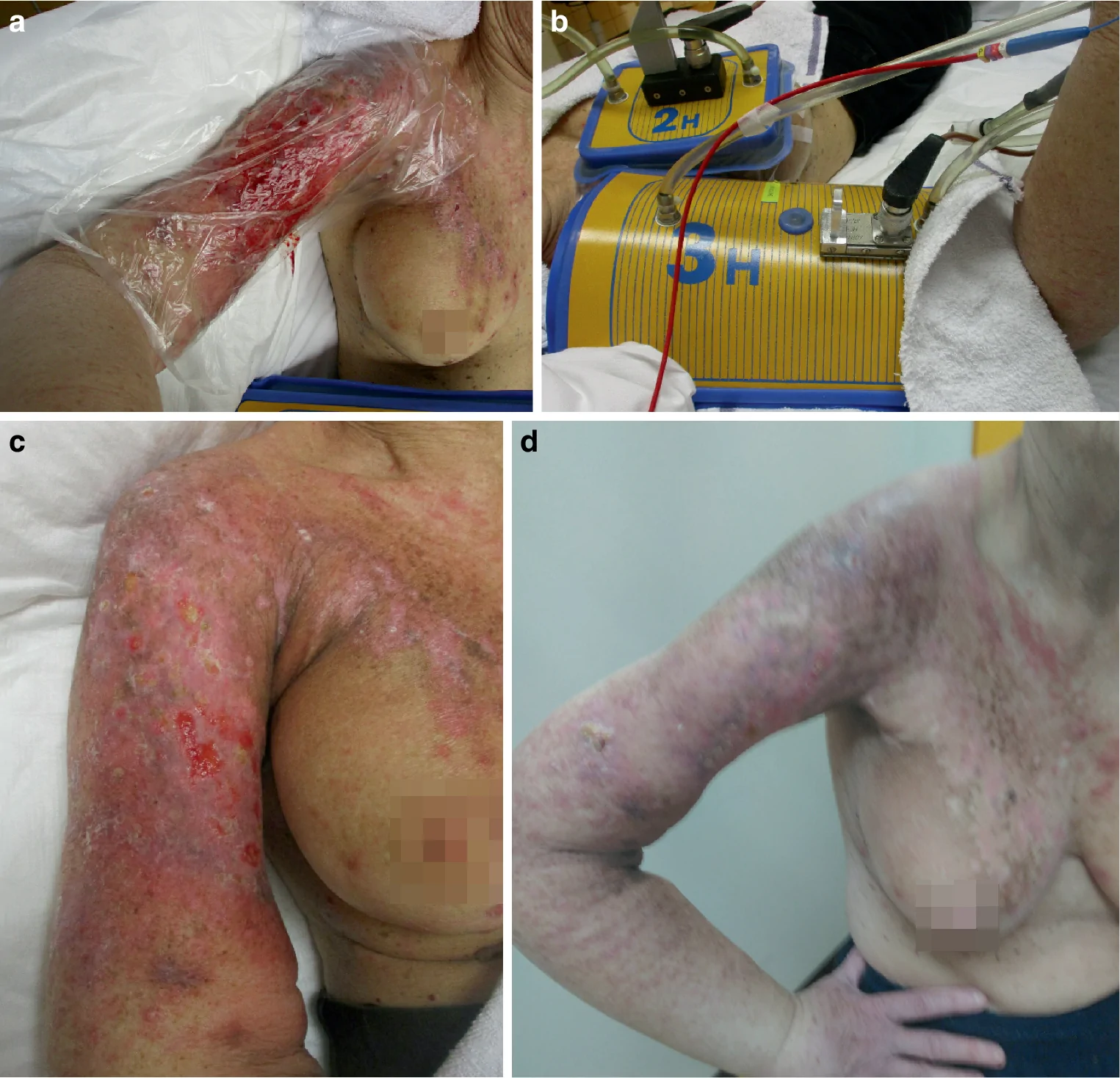
The right arm and right breast before start of radiotherapy and hyperthermia treatment (**a**), hyperthermia treatment setup (**b**), at the day of last treatment (**c**), and 1 month after the final fraction (**d**)

A 72-year old woman presented with extensive cutaneous lesions of the right thorax wall and right arm. Four years earlier she was diagnosed with metastatic breast cancer (ER− PR− HER2+) with cutaneous metastases and a single pulmonary lesion. First-line treatment was chemotherapy with a combination of docetaxel, pertuzumab, and trastuzumab; thereafter, she was treated with radiotherapy on the right breast and shoulder with 5 fractions of 6 Gy on the breast and skin. About half a year later, she developed progression of lymphangitic carcinomatosis, especially in the right thorax, shoulder, and arm, and treatment with trastuzumab–emtansine (T-DM1) was initiated. Again half a year later, she showed progression of cutaneous metastases and treatment was switched to T‑deruxtecan. About a year later again cutaneous lesions in the right upper arm were observed for which she received one fraction of 8 Gy. The treatment courses over time are shown in Fig. 1. Shortly after the lesions started to bleed and itch, impacting quality of life. The patient was referred for palliative radiotherapy with the aim to stop itching and bleeding of the right upper arm, right shoulder and cranial of the right breast (Fig. 2a). Superficial hyperthermia was added to the treatment to enhance the radiotherapy effect.

The treatment regimen included a total of five therapy sessions scheduled once a week, while T‑deruxtecan was temporarily halted during radiation treatment. Each session started with superficial hyperthermia treatment, in which a targeted temperature of at least 41 °C for 60 min, followed within 5 min by 4 Gy radiotherapy dose. The treatment courses over time are illustrated in Fig. 1. This scheme is comparable to the treatment schema proposed by Notter et al. for macroscopic breast cancer tumors [11]. The ultrashort time interval maximizes the radiosensitizing effect of hyperthermia, which is estimated to enhance the radiation dose by a factor of 1.34 when assuming simultaneous irradiation and hyperthermia treatment and a median temperature of 41.5 °C during 60 min [12]. The total radiation dose to the target area in this patient is therefore estimated at 5.4 Gy per fraction instead of 4 Gy. With the assumption of an α/β ratio of 3.7 Gy according to the FAST-Forward trial [13], the estimated EQD2 increased from 27.0 Gy without hyperthermia to 31.9 Gy with hyperthermia. It is important to note that the mentioned numbers are estimations and not verified in a control group. The target depth was from skin up to 1–2 cm beneath the skin and clinical guidelines recommend invasive thermometry for lesions exceeding 1 cm depth [10]. However, in this case no interstitial temperature measurements were possible because of the sensitive and bleeding skin.

The patient completed all five superficial hyperthermia sessions using the radiative 434 MHz Alba ON 4000D hyperthermia system (Medlogix Srl, Rome, Italy) using two flexible microstrip applicators (Istok, Fryazino, Russia) [14]. A 3H applicator with an effective field size of 18 × 24 cm was positioned around the right upper arm, while an 2H applicator with an effective field size of 9 × 12 cm was located on the right breast (Fig. 2b).

Table 1 shows the median skin temperature (T50) and the median cumulative equivalent minutes at 43 °C (CEM43T50) per applicator [15]. Bleeding of the skin decreased during therapy; the patient continued to experience itching because of the dry skin and increased edema of the right lower arm. Four weeks after the final treatment, all wounds were closed and the skin was no longer itchy (Fig. 2d). Edema of the right lower arm remained, and the patient was referred for edema therapy. The patient experienced fatigue; however, overall she tolerated treatment very well. The patient visited the treating physician weekly during the treatment course and all complaints were questioned extensively. Also during hyperthermia sessions, the patient was frequently asked about pain or other discomfort by specialized hyperthermia radiotherapists. She was able to continue with voluntary work and daily walks. New cutaneous lesions were observed at the border of the radiation field after approximately 2 months and systemic therapy was switched to tucatinib–capecitabine–trastuzumab. The patient passed away 6 months after completing the series of combined radiotherapy and hyperthermia due to disease progression outside the radiation field.

**Table 1:** Achieved temperatures during superficial hyperthermia treatment measured on the skin.

		Session				
Location	Parameter	#1	#2	#3	#4	#5
Right upper arm	T50 (°C)	41.1	41.7	41.4	41.4	41.4
	CEM43T50 (min)	5.78	9.83	7.62	10.05	7.19
	Maximum power (W)	66	100	98	100	100
	Maximum water bolus temperature (°C)	43.5	43.0	44.0	44.7	44.3
Right breast	T50 (°C)	41.5	41.9	41.8	41.8	41.6
	CEM43T50 (min)	6.91	13.03	10.39	10.96	8.19
	Maximum power (W)	58	58	98	84	90
	Maximum water bolus temperature (°C)	42.5	42.5	43.5	44.0	42.7
Time between end of hyperthermia and start of radiotherapy (min)		26	26	20	37	23

## Discussion and conclusion

Cutaneous metastases of breast cancer are the most common skin metastases in women and tend to metastasize to the chest wall. Other locations like upper extremities, the abdomen, and back are less frequently observed. Treatment of cutaneous metastases is dependent on the extent of the disease and hormonal status. Local control can be achieved with radiotherapy; however, in previous irradiated areas, the delivered dose is limited to avoid radiation toxicity [16]. Hyperthermia is an effective valuable option for these patients to boost radiotherapy dose without increasing radiotherapy side effects [6, 17].

The importance of high temperatures and short intervals between radiotherapy and hyperthermia has been shown for breast cancer, melanoma, and other types of cancer, resulting in improved local control [11, 12, 18, 19]. In superficial located tumors (< 3 cm), significant temperature dose–effect associations were observed when temperatures were measured in the target area with interstitial thermometry [9, 17, 18]. For instance, a best session CEM43T50 (median cumulative equivalent minutes at 43 °C) above 7.2 min was found to be associated with improved local control [9, 17, 18]. The best session CEM43T50 in our series exceeded this 7 min level and was expected to achieve good therapeutic effect (Table 1). Superficial hyperthermia guidelines recommend minimum tumor temperatures ≥ 40 °C and median temperature T50 ≥ 41 °C [10]; our reported median temperatures T50 all exceed 41 °C (Table 1).

However, correlations between skin temperature and outcome are not reported in the literature for cutaneous lesions [9]. The reason is that the measured skin temperatures are often average values between the true skin temperatures and the often much cooler water bolus temperature [20]. Nevertheless, in this case the maximum set temperature of the water bolus varied between 42.5 °C and 44.7 °C (Table 1) during and between treatment sessions. These temperatures are close to the target temperatures; hence, skin measurements are likely to give a correct reflection of the invasive temperature [21].

In conclusion, the patient presented in this short communication was successfully treated with palliative radiotherapy combined with hyperthermia for challenging bleeding cutaneous breast cancer metastases in a previously irradiated area which had a large impact on the quality of life. The treatment schedule was well-tolerated and the skin recovered within 1 month after therapy completion. Patients with a clinical presentation similar to the case presented are suggested to be referred to specialized hyperthermia centers, which will generate more experience and evidence for treating patients with (bleeding) cutaneous metastases.

## Data Availability

Available upon reasonable request.

## Abbreviations

CEM43T50: Median cumulative equivalent minutes at 43 °C
ER-: Estrogen receptor negative
Gy: Gray
HER+: human epidermal growth factor receptor 2 positive
HT: Hyperthermia
PR-: Progesterone receptor negative
T50: Median temperature
